# Adherence to the Mediterranean diet is associated with lower cancer-related fatigue: a cross-sectional analysis from NHANES 2017–2020

**DOI:** 10.3389/fnut.2025.1506055

**Published:** 2025-03-19

**Authors:** Xueqin Xia, Xuehua Cao, Chen Gong, Yi Liu, Xiaoyuan Zhang, Limei Liao

**Affiliations:** ^1^Department of Obstetrics and Gynecology, Sichuan Provincial People’s Hospital, University of Electronic Science and Technology of China, Chengdu, China; ^2^School of Medicine, University of Electronic Science and Technology of China, Chengdu, China; ^3^Department of Emergency Intensive Care Unit, Sichuan Provincial People’s Hospital, University of Electronic Science and Technology of China, Chengdu, China

**Keywords:** Mediterranean diet, cancer-related fatigue, NHANES, alternate Mediterranean diet score, dietary pattern

## Abstract

**Background and objectives:**

Cancer-related fatigue is a common and distressing symptom experienced by cancer patients, which may persist from the time of diagnosis to the end of life. This fatigue negatively affects patients’ physical, emotional, and cognitive well-being. Nutrition plays a key role in managing cancer-related fatigue, and recently, the Mediterranean diet has gained attention as a potential intervention. The present study uses data from the National Health and Nutrition Examination Survey (NHANES) to investigate the association between cancer-related fatigue and the Mediterranean diet.

**Methods:**

Data from the NHANES 2017–2020.03 cycle were selected for this cross-sectional study. The Alternative Mediterranean Diet Adherence (AMED) score was used to evaluate the participants’ adherence to the Mediterranean diet. AMED scores were calculated based on data from 24-h dietary recall interviews conducted on both day one and day two. Multiple linear regression modeling was used to explore the association between AMED scores and cancer-related fatigue, as well as the relationship between AMED scores and fatigue in the general population.

**Results:**

A total of 6,413 adults aged 20 years and older were included in the study, with 707 identified as cancer patients. There was a noteworthy inverse relationship found between AMED scores and fatigue, which was more pronounced in cancer patients: *β* = −0.121, 95% CI: −0.172, −0.071 (*p* < 0.001) in the unadjusted model. This correlation remained significant after adjusting for all variables in model 3: *β* = −0.074, 95% CI: −0.127, −0.021 (*p* = 0.007). A significant dose-dependent relationship was found when AMED scores were expressed in quartiles, with a more pronounced negative association as AMED increased across all models (*p* for trend <0.05). In the cancer population, the analysis of individual nutrients and fatigue revealed that alcohol was significantly negatively associated with cancer-related fatigue in all models, particularly in the unadjusted model: *β* = −0.710, 95% CI: −1.058, −0.362 (*p* < 0.001). Subgroup analyses indicated that diabetes, education level and type of cancer had a significant effect on the relationship between AMED and fatigue, with interaction *p*-values of 0.010, 0.023 and 0.049, respectively.

**Conclusion:**

The present study suggests that higher adherence to the Mediterranean diet may contribute to reduce fatigue, especially in cancer patients; however, further research is necessary to validate this correlation.

## 1 Introduction

Fatigue is one of those most prevalent symptoms among cancer patients, and it can occur both during treatment and in survivors. Cancer-related fatigue (CRF) is defined as a state of profound and prolonged physical, emotional, and/or mental exhaustion, triggered by cancer or its treatment, that is disproportionate to an individual’s recent activity level and interferes with daily functioning (1, 2). CRF can impact patients’ physical health, psychological well-being, social interactions, and all aspects of economic and professional life, including that of their caregivers, greatly reducing their quality of life (3, 4). The prevalence of CRF varies widely, from 11% (5) to 99% (6) depending on the type of cancer, treatment and other factors (1, 7). In a meta-analysis including 71,568 cancer patients, the overall prevalence of CRF was found to be as high as 49% (8). This means that nearly half of cancer patients may experience CRF. Given its significant impact on cancer patients, it is necessary to better understand the modifiable factors that may reduce CRF.

The pathogenesis of CRF remains unclear, potentially linked to cancer treatment (9), peripheral immune activation and inflammatory response (10), muscle metabolic disorders (11), nutrition (12), anemia (13), central nervous system dysfunction (14), and sleep disorders or depression (15). Clinical practice recommends exercise, cognitive-behavioral therapy, and mindfulness-based psychological interventions for managing CRF, but the effectiveness of medication or other psychological interventions has not been determined (16). However, there are also certain obstacles to the implementation of exercise or psychological interventions such as cognitive-behavioral therapy (CBT). Many patients generally doubt the efficacy of exercise in mitigating fatigue, while physical and health constraints, insufficient time, interest, motivation, or facilities, along with inadequate support from family and friends, can all impede the execution of exercise interventions (13). Although cognitive-behavioral therapy may be beneficial, it has not yet been determined which patients benefit from which type of psychosocial intervention (17). At the same time, many psychological therapies require specialized training and qualified medical personnel to implement, and patients need to visit psychological and social service providers specialized in cancer care (13).

Nutrition is also an important modifiable factor contributing to CRF, potentially alleviating fatigue while posing a lower burden than other interventions (18). Previous studies have demonstrated its promising effectiveness. In a prospective cohort study exploring the relationship between dietary quality and fatigue, researchers included 707 women with breast cancer. Dietary data were collected 30 months after diagnosis, while fatigue assessments were conducted 41 months after diagnosis. The study demonstrated that survivors with better-quality diets exhibited significantly lower total fatigue scores (4.1vs.4.8, *p* < 0.05) (19). The study utilized the Healthy Eating Index 2010 (HEI-2010) to assess diet quality; high-quality diets were characterized by a higher intake of fruits, vegetables, legumes, whole grains, protein, healthy fats, and dairy products, as well as a lower intake of fatty acids, refined grains, sodium, and empty calories (20). The Mediterranean diet has also been identified as high-quality dietary pattern characterized by similar wholesome food components, notably a diet abundant in fruits and vegetables, nuts, whole grains, legumes, fish, and olive oil (21). A recent systematic review confirmed the safety and feasibility of the Mediterranean diet in adults with cancer (22). Previous studies have shown that the Mediterranean diet has weight loss effects by reducing energy intake, and the decrease in weight gain is associated with improved fatigue levels (23). The Mediterranean diet’s components, notably omega-3, antioxidants, and dietary fiber, may contribute to alleviating fatigue through anti-inflammatory pathways (10, 24). Furthermore, the Mediterranean diet has shown promise in the treatment of other health conditions, such as depression (25, 26). For instance, a cross-sectional survey that included 11,769 adults found that greater adherence to the Mediterranean diet was associated with 40–45% decreased odds of moderate to severe depressive symptoms (25).

Previous studies have investigated the effectiveness of Mediterranean diet intervention in CRF management. In a study, 23 prostate cancer patients were randomly assigned to either 12 weeks of routine care or the Mediterranean-style dietary pattern (MED-diet) group. The results of this study showed that the MED-diet group achieved significant improvements in CRF scores at 8 and 12 weeks, along with a notable enhancement in quality of life scores at 12 weeks (27). Similarly, in another study by Amber S. Kleckner and colleagues, 33 women with breast cancer were randomly assigned to either the usual care group or the Mediterranean Diet (MedDiet) group, with the latter group receiving an eight-week intervention. Compared to usual care, the Mediterranean diet demonstrated a small to moderate effect on fatigue in these breast cancer patients at the 4th and 8th weeks (28). The effects of the Mediterranean diet on CRF have been studied, however, most of these studies were pilot studies with small sample sizes, and there are not many large-scale studies available. Patients with breast cancer and prostate cancer are the primary subjects of study; hence, the generalizability of the findings is constrained by the paucity of studies conducted on more varied and inclusive populations. The National Health and Nutrition Examination Survey (NHANES) is a valuable resource that can be used to address this research gap. NHANES is a collection of nationally representative health survey data intended to track the state of health in the United States population. The data encompasses demographics, dietary, examination, laboratory, and questionnaire information. NHANES is a representative sample of American citizens because it uses a sophisticated multi-stage probability sampling design. Therefore, the purpose of this study is to explore the relationship between the Mediterranean Diet and CRF using data from NHANES.

## 2 Methods

### 2.1 Study population and design

NHANES provided the data for this cross-sectional survey, and 15,560 respondents in all were chosen between 2017 and 2020.03. Exclusion criteria included: (1) Being under 20 years old; (2) Lack of information on cancer diagnosis history; (3) Absence of fatigue data; (4) Absence of dietary consumption data. The NHANES study was approved by the Ethics Review Committee of the National Center for Health Statistics, with all participants providing written informed consent. The NHANES data is accessible at the following website: http://www.cdc.gov/nchs/nhanes.htm.

### 2.2 Assessment of alternate Mediterranean diet (AMED) score

Participants’ Mediterranean diet adherence was assessed using the Alternative Mediterranean Diet (AMED) score (29). Mean dietary intake over 2 days was assessed using data from two 24-h dietary recall interviews of NHANES. Three to ten days after the first dietary recall interview, which is conducted in person at the Mobile Examination Center (MEC), the second interview is conducted over the phone. Dietary intake for each participant was assessed by taking the average of two separate 24-h recalls. Adherence to the Mediterranean Diet was calculated in two steps. (1) Link the 24-h dietary recall data to the United States Department of Agriculture (USDA) Food Patterns Equivalents Databases to convert different foods and beverages into equal amounts of food pattern components. (2) Adherence to MED was assessed using the alternative Mediterranean Diet (AMED) index (30, 31). The AMED comprises nine components: fruits, vegetables, legumes, nuts, whole grains, red and processed meats, fish, alcohol, and the ratio of monounsaturated to saturated fats. For a given food component, participants scored 1 point if intake was above the median, except for red/processed meat and alcohol. Participants scored 1 point if intake of red/processed meat was below the median, and 1 point if intake of alcohol was moderate (defined as 10–25 grams per day for males and 5–15 grams per day for females) (32). The total AMED score varied from 0 to 9, with elevated scores indicating enhanced adherence to the Mediterranean diet.

### 2.3 Assessment of fatigue

Scales specifically designed to assess cancer-related fatigue are not available in the NHANES database. However, fatigue can be assessed using individual survey item from this database. Specifically, fatigue can be measured by a single item in the Patient Health Questionnaire Depression Scale (PHQ-9) (33). This question (DPQ040) asks participants, “Over the last 2 weeks, how often have you been bothered by the following problem: feeling tired or having little energy?” Response options ranged from “not at all” (0) to “nearly every day” (3), with “several days” (1) and “more than half the days” (2) in between. The scores therefore range from 0 to 3, with higher scores indicating more severe fatigue, which has been used in prior study (34).

Single-item fatigue measures have been used in several studies in both the general population and cancer patients. For example, in a general population study, the question “How tired are you right now?” was used to assess fatigue on a scale from 1 (“not at all”) to 10 (“extremely tired”). Ratings from the six fatigue subscales of the Dutch version of the Profile of Mood States (POMS) (35) were compared, revealing strong correlations between the single-item question and multidimensional fatigue ratings. This study demonstrated that single-item fatigue measures can effectively capture the essence of fatigue (36). In addition, the Zung Self-Rating Depression Scale (ZSDS) (37) includes a fatigue item that has been used to screen for cancer-related fatigue (38). The item assesses the frequency of fatigue using a four-point scale: none/occasionally, sometimes, often, and always. When compared with the Fatigue Symptom Inventory (FSI) (39), the Zung scale fatigue item had a sensitivity of 78.95% and a specificity of 87.88% while using a score of 3 as the cut-off. This further supports the use of single-item measures in fatigue research.

CRF is a subjective experience that is most effectively measured by self-report (40). For this study, the fatigue item from the PHQ-9 was selected as the primary measure of fatigue to assess both the presence and severity of fatigue in participants.

### 2.4 Assessment of covariates

Based on existing literature, we selected covariates known or hypothesized to influence the relationship between Mediterranean diet and cancer-related fatigue (40–46). Included covariates: race, gender, education, age, body mass index (BMI), hypertension, health insurance, marital status, diabetes, physical activity, alcohol use, and sleep disorders. Including these variables in our analysis allowed us to control for potential confounders and better isolate the effect of the Mediterranean diet on fatigue. Our goal in controlling for these factors was to increase the validity and accuracy of our results and provide a more trustworthy evaluation of the relationship between fatigue and diet adherence in our study sample.

Specifically, gender included male and female. Race included Mexican American, other Hispanic, non-Hispanic Black, non-Hispanic White, and other races (including multi-racial). Education was categorized into ‘Less than 9th grade’, ‘9-11th grade’, ‘High school grade /GED or equivalent’, ‘Some college or AA degree’, and ‘College graduate or above’. Marital status included ‘Married/Living with Partner’, ‘Widowed/Divorced/Separated’, and ‘Never married’. According to participant’s BMI they were categorized into obesity (BMI ≥ 30.0 kg/m^2^), overweight (25 kg/m^2^ ≤ BMI < 30 kg/m^2^), normal weight (18.5 kg/m^2^ ≤ BMI < 25.0 kg/m^2^), or underweight (BMI < 18.5 kg/m^2^) (47). Hypertension was defined by the question of ‘Ever told you had high blood pressure?’ (Yes/No). Diabetes was defined by the question of ‘Doctor told you have diabetes?’ (Yes/No). The question “Ever told by the doctor you have a sleep disorder?” (Yes/No) was used to evaluate sleep disorders. Alcohol use was categorized into non-drinker (less than 12 cups in their lifetime), or drinker (48). Health insurance information was obtained by asking the question, “Are you covered by health insurance or another type of health care plan?” (Yes/No). Physical activity (PA) was measured in metabolic equivalent tasks (METs) and calculated using the following formula: physical activity (MET· min/week) = recommended MET × duration of activity (min/day) × frequency of activity (less than days/week). The physical activity levels were classified into three categories: low (600 MET-min/week), moderate (between 600 and 1,500 MET-min/week), and high (more than 1,500 MET-min/week) (49).

### 2.5 Statistical analyses

This cross-sectional study aimed to investigate the relationship between the AMED and CRF. Data were collected from the 2017–March 2020 NHANES cycle, using the recommended weighting methods to ensure accuracy and representativeness. Specifically, this study applied the weights for the 24-h dietary recall on the second day (WTDR2DPP) to correct for sampling bias and enhance the generalizability of the results.

Participants were grouped based on cancer status (with or without cancer), and their AMED scores were divided into quartiles to better evaluate the impact on fatigue both across the entire population and within each group. Baseline characteristics were described using descriptive statistics: continuous variables were presented as mean ± standard deviation (Mean ± SD), whereas non-normally distributed continuous variables were reported as median (M) and interquartile range (IQR: Q1, Q3). Categorical variables were represented as frequency and percentage (*n*, %).

To examine the association between AMED scores and cancer-related fatigue, we employed both linear and non-linear statistical models. Specifically, we used weighted multiple linear regression analysis to assess the linear relationship between AMED adherence and fatigue. Three regression models were developed: Model 1 did not adjust for any variables; Model 2 adjusted for key demographic factors, including gender, race, and age; and Model 3 for adjusted race, gender, education, age, body mass index (BMI), hypertension, health insurance, marital status, diabetes, physical activity, alcohol use, and sleep disorders. These models were used to assess the trend of fatigue across quartiles of AMED scores, with a trend test employed to evaluate the linearity of this relationship. To explore potential non-linear relationships between AMED scores and fatigue, we employed a weighted generalized additive model (GAM) with smooth curve fitting.

Furthermore, we conducted subgroup analyses of participants in the cancer group to explore whether there were differences in the relationship between AMED adherence and cancer-related fatigue across demographic and clinical subgroups. Interaction effects were performed to assess whether the effect of AMED adherence on fatigue differs significantly across these subgroups. Statistical significance was defined as a two-sided *p*-value <0.05 for all analyses.

All statistical analyses were conducted using R software (version 4.2.0, The R Foundation^1^) and EmpowerStats^2^ (X&Y Solutions, Inc., Boston, MA).

## 3 Results

### 3.1 Baseline characteristics

**Figure 1:**
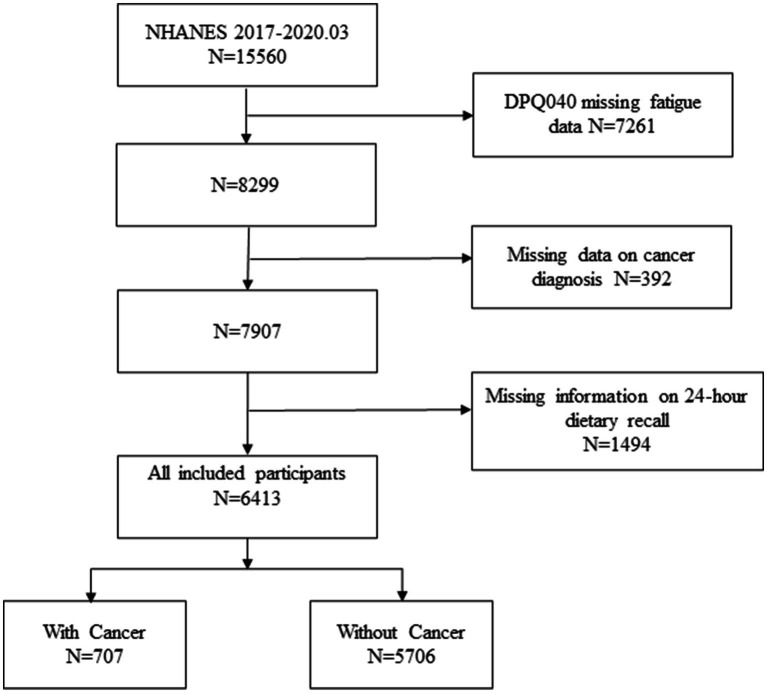
The flowchart of participants.

Of the 15,560 individuals identified in the search results, we excluded 7,261 due to missing fatigue data, 392 due to missing cancer diagnoses, and 1,494 due to incomplete AMED calculations. The final sample comprised 6,413 eligible participants (Figure 1). The mean (SD) age of the participants was 48.46 ± 17.28 years, with 51.83% being female and 63.38% identified as Non-Hispanic White. Of these, 707 (11.02%) were cancer patients whose cancer diagnoses included the 27 cancer diagnoses listed by NHANES, as well as others and those who did not know their diagnosis. 71 had two cancers, 14 had three, and another 3 had more than three or more cancers, the exact numbers and diagnoses are listed in Supplementary Table S1. Statistical differences were observed between cancer and non-cancer group in terms of cancer diagnosis, AMED score, age, gender, race, education, marital status, BMI, alcohol consumption, hypertension, diabetes, health insurance, MET, and sleep disorders (*p* < 0.05). Fatigue scores did not differ significantly between cancer and non-cancer groups (median [IQR]: 1 [0–1] for both; Table 1). However, a statistically significant discrepancy was observed in the distribution of fatigue scores between the two groups χ^2^ (3) = 13.99, *p* = 0.003. Further stratified analysis revealed significant differences in the proportions of fatigue scores between the two groups across various strata (0, 1, 2, 3). Specifically, statistically significant differences were found in score groups 1 and 3, with the cancer group exhibiting a higher proportion of participants reporting almost daily fatigue (score of 3) compared to the non-cancer group (12.02% vs. 8.13%) (*p* < 0.001). Additionally, a significantly higher proportion of participants in the non-cancer group had a fatigue score of 1 compared to the cancer group (33.42% vs. 29.56%) (*p* = 0.039). The remaining score strata showed no statistically significant differences (*p* > 0.05) (Supplementary Table S2).

**Table 1:** Demographic characteristics of participants, weighted.

	Total (*n* = 6,413)	Cancer (*n* = 707)	Non-cancer (*n* = 5,706)	*p*-value
AMED score	3.458 ± 1.395	3.664 ± 1.358	3.432 ± 1.398	< 0.001
Alcohol	0.078 ± 0.209	0.073 ± 0.197	0.079 ± 0.211	0.479
Monounsaturated to saturated fat ratio	0.516 ± 0.375	0.518 ± 0.382	0.516 ± 0.374	0.907
Red and processed meat	0.447 ± 0.386	0.502 ± 0.388	0.439 ± 0.386	< 0.001
Fish	0.157 ± 0.271	0.188 ± 0.290	0.154 ± 0.268	0.001
Nuts	0.400 ± 0.399	0.442 ± 0.398	0.395 ± 0.398	0.003
Legumes	0.273 ± 0.341	0.221 ± 0.313	0.280 ± 0.344	0.000
Whole grains	0.493 ± 0.402	0.537 ± 0.385	0.488 ± 0.403	0.002
Vegetables	0.617 ± 0.372	0.635 ± 0.377	0.615 ± 0.372	0.171
Fruit	0.476 ± 0.409	0.548 ± 0.409	0.466 ± 0.408	<0.001
Age	48.458 ± 17.276	63.068 ± 14.284	46.603 ± 16.731	<0.001
Gender				< 0.001
Male	48.173	41.585	49.01	
Female	51.827	58.415	50.99	
Race				< 0.001
Mexican American	7.928	2.929	8.563	
Other Hispanic	7.675	2.672	8.311	
Non-Hispanic White	63.377	82.478	60.951	
Non-Hispanic Black	11.291	6.771	11.865	
Other Race – Including Multi-Racial	9.728	5.15	10.31	
Education				0.003
Less than 9th grade	2.611	1.546	2.747	
9-11th grade (12th grade with no diploma)	6.567	5.419	6.713	
High school graduate/GED or equivalent	26.493	23.092	26.925	
Some college or AA degree	30.484	30.033	30.541	
College graduate or above	33.829	39.909	33.056	
Not record	0.016		0.018	
Marital status				< 0.001
Married/Living with Partner	62.992	65.032	62.733	
Widowed/Divorced/Separated	17.85	28.703	16.472	
Never married	19.127	6.117	20.779	
Not record	0.031	0.148	0.017	
BMI (kg/m^2^)				0.009
Underweight	1.184	1.031	1.203	
Normal weight	24.824	20.199	25.411	
Over weight	31.128	36.056	30.502	
Obesity	42.455	42.317	42.472	
Not record	0.409	0.397	0.411	
Alcohol use				< 0.001
Non-drinker	39.916	46.649	39.06	
Drinker	53.161	48.029	53.813	
Not record	6.924	5.321	7.127	
Hypertension				< 0.001
Yes	32.794	48.587	30.788	
No	67.071	51.204	69.086	
Not record	0.135	0.209	0.126	
Diabetes				< 0.001
Yes	11.543	18.729	10.631	
No	86.08	77.99	87.108	
Not record	2.376	3.281	2.262	
Fatigue (median, IQR)	1 (0,1)	1 (0,1)	1 (0,1)	0.127
Fatigue score (*n*, %)				0.003
0		351 (49.65)	2,824 (49.47)	
1		209 (29.56)	1908 (33.42)	
2		62 (8.77)	512 (8.97)	
3		85 (12.02)	462 (8.13)	
Health Insurance				< 0.001
Yes	87.244	96.921	86.016	
No	12.611	2.895	13.845	
Not record	0.145	0.184	0.14	
Physical activity (MET, min/week)				< 0.001
Low	13.18	12.84	13.223	
Moderate	20.669	24.912	20.13	
High	46.852	34.706	48.394	
Not record	19.299	27.542	18.252	
Sleep disorder				< 0.001
Yes	31.055	38.577	30.1	
No	68.932	61.423	69.886	
Not record	0.013		0.014	

CI, confidence interval; AMED, Alternative Mediterranean Diet; MET, Metabolic Equivalent Task; BMI, Body Mass Index; *p* < 0.05 presents significant difference.

### 3.2 Association between AMED score and fatigue in cancer and non-cancer groups

**Figure 2:**
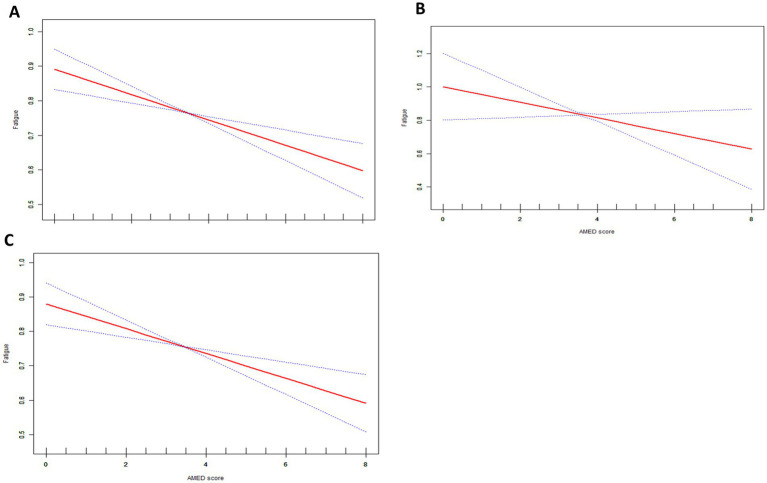
The association between AMED scores and fatigue red line represents the smoothed curves fit between variables. The blue line represents the 95% confidence interval. **(A)** Total population all covariates in Table 1 were adjusted. **(B)** Cancer group, all covariates in Table 1 except cancer were adjusted. **(C)** Non-Cancer group, all covariates in Table 1 except cancer were adjusted.

We investigated the relationship between participants’ overall fatigue and AMED scores, as well as those with and without cancer. Three weighted models were utilized as well for the statistical assessments. We further divided the AMED scores by quartiles to investigate the association between AMED and fatigue in more detail (Table 2). Weighted generalized additive models and smoothed curve fitting were employed to visually evaluate the connection between the variables (Figure 2). Figure 2 illustrates a linear negative correlation between AMED scores and fatigue, consistently observed across the overall population as well as in both cancer and non-cancer cohorts.

**Table 2:** Associations between AMED (Total/Quartiles) score and fatigue in participants with and without cancer, weighted.

Variable	Cancer (*n* = 707) *β* (95%CI), *p*-value	non-cancer (*n* = 5,706) *β* (95%CI), *p*-value	Total (*n* = 6,413) *β* (95%CI), *p*-value
Model 1^a^			
AMED score	−0.121 (−0.172, −0.071) <0.001	−0.068 (−0.085, −0.052) <0.001	−0.074 (−0.090, −0.058) <0.001
AMED score quartile			
Q1 (reference)	NA	NA	NA
Q2	−0.165 (−0.390, 0.060) 0.151	−0.096 (−0.163, −0.028) 0.005	−0.100 (−0.165, −0.035) 0.002
Q3	−0.377 (−0.590, −0.163) <0.001	−0.113 (−0.180, −0.047) <0.001	−0.141 (−0.205, −0.077) <0.001
Q4	−0.414 (−0.626, −0.202) <0.001	−0.272 (−0.338, −0.206) <0.001	−0.284 (−0.347, −0.221) <0.001
*p* for trend	<0.001	<0.001	<0.001
Model 2^b^			
AMED score	−0.120 (−0.171, −0.070) <0.001	−0.081 (−0.098, −0.064) <0.001	−0.085 (−0.101, −0.069) <0.001
AMED score quartile			
Q1 (reference)	NA	NA	NA
Q2	−0.170 (−0.397, 0.056) 0.141	−0.107 (−0.174, −0.040) 0.002	−0.112 (−0.177, −0.048) <0.001
Q3	−0.383 (−0.598, −0.169) <0.001	−0.132 (−0.199, −0.065) <0.001	−0.158 (−0.222, −0.094) <0.001
Q4	−0.417 (−0.631, −0.202) <0.001	−0.318 (−0.385, −0.251) <0.001	−0.325 (−0.389, −0.261) <0.001
*p* for trend	<0.001	<0.001	<0.001
Model 3^c^			
AMED score	−0.074 (−0.127, −0.021) 0.007	−0.035 (−0.052, −0.017) <0.001	−0.038 (−0.054, −0.022) <0.001
AMED score quartile			
Q1 (reference)	NA	NA	NA
Q2	−0.239 (−0.463, −0.015) 0.037	−0.078 (−0.142, −0.014) 0.018	−0.086 (−0.148, −0.024) 0.006
Q3	−0.305 (−0.523, −0.087) 0.006	−0.065 (−0.130, 0.000) 0.051	−0.081 (−0.143, −0.019) 0.011
Q4	−0.316 (−0.536, −0.096) 0.005	−0.152 (−0.220, −0.084) <0.001	−0.162 (−0.227, −0.097) <0.001
*p* for trend	0.011	<0.001	<0.001

CI, confidence interval; AMED, Alternative Mediterranean Diet; NA, not applicable. ^a^Model 1 was non-adjusted model; ^b^Model 2 was adjusted for gender, race, age; ^c^Model 3 was adjusted for the following variables: gender, race, age, physical activity (MET), health insurance, sleep disorders, diabetes, education, hypertension, marital status, BMI, and alcohol use. *p* < 0.05 presents significant difference.

#### 3.2.1 Cancer group

In the cancer group (*n* = 707), higher AMED scores were consistently associated with lower levels of fatigue across all models. In the unadjusted model (Model 1), the *β* coefficient for total AMED score was −0.121 (95% CI: −0.172, −0.071), indicating a significant negative association between AMED and fatigue (*p* < 0.001). After adjusting for gender, age, and race in Model 2, the effect size slightly decreased, with a *β* of −0.120 (95% CI: −0.171, −0.070, *p* < 0.001). In the fully adjusted model (Model 3), which controlled for all variables in Table 1 (e.g., BMI, education, hypertension, alcohol consumption, physical activity, and health insurance), the association remained significant, with a *β* of −0.074 (95% CI: −0.127, −0.021, *p* = 0.007).

When AMED score was analyzed by quartiles, a clear dose–response relationship was observed. Participants in the highest quartile (Q4) of AMED score exhibited the greatest reduction in fatigue levels compared to the reference group (Q1), with a *β* of −0.414 (95% CI: −0.626, −0.202, *p* < 0.001) in Model 1. This trend persisted across Models 2 and 3, with significant results in each case. The *p* for trend value was <0.001 in both models, while it was 0.011 in Model 3.

#### 3.2.2 Non-cancer group

Among the non-cancer participants (*n* = 5,706), a similar negative association between AMED score and fatigue was observed. In Model 1, the *β* for total AMED score was −0.068 (95% CI: −0.085, −0.052, *p* < 0.001), suggesting a moderate inverse relationship. This association strengthened after adjusting for age, gender, and race in Model 2 (*β* = −0.081, 95% CI: −0.098, −0.064, *p* < 0.001). In Model 3, the effect size decreased after further adjustment for all covariates (*β* = −0.035, 95% CI: −0.052, −0.017, *p* < 0.001). Quartile analysis showed a similar trend in the non-cancer group, where participants in Q4 had significantly lower fatigue scores compared to those in Q1. In the fully adjusted model (Model 3), the *β* for Q4 was −0.152 (95% CI: −0.220, −0.084, *p* < 0.001), and the *p* for trend was also significant (*p* < 0.001). The same trend was observed in both Model 1 and Model 2, with *p* for trend values of <0.001, indicating a linear association between higher AMED quartiles and reduced fatigue.

#### 3.2.3 Total population

In the combined analysis of the total population (*n* = 6,413), the overall findings were consistent. In Model 1, the *β* for total AMED score was −0.074 (95% CI: −0.090, −0.058, *p* < 0.001), with similar results in Models 2 and 3. The fully adjusted model (Model 3) demonstrated a significant inverse association, with a *β* of −0.038 (95% CI: −0.054, −0.022, *p* < 0.001). Quartile analysis in the total population also revealed a significant dose–response relationship in all three models. In Model 3, participants in Q4 showed the greatest reduction in fatigue scores (*β* = −0.162, 95% CI: −0.227, −0.097, *p* < 0.001), and a significant linear trend was observed across quartiles (*p* for trend <0.001).

### 3.3 Association between AMED components and fatigue

#### 3.3.1 Cancer group

In the cancer population, various AMED components were significantly linked to fatigue (Table 3). Alcohol consumption exhibited the strongest inverse association across all models, especially in Model 1 (*β* = −0.710, 95% CI: −1.058, −0.362, *p* < 0.001). Nuts, fruit, and the monounsaturated to saturated fat ratio also demonstrated significant inverse associations with fatigue in Models 1 and 2. Red and processed meat showed a significant association only in Model 2 (*p* = 0.049).

**Table 3:** Association between AMED components and fatigue weighted.

	Cancer (*n* = 707) *β* (95%CI), *p*-value	Non-cancer (*n* = 5,706) *β* (95%CI), *p*-value	Total (*n* = 6,413) *β* (95%CI), *p*-value
Model 1^a^			
Alcohol	−0.710 (−1.058, −0.362) <0.001	−0.148 (−0.257, −0.038) 0.008	−0.204 (−0.309, −0.099) <0.001
Monounsaturated to saturated fat ratio	−0.226 (−0.406, −0.045) 0.015	−0.174 (−0.236, −0.112) <0.001	−0.180 (−0.238, −0.122) <0.001
Red and processed meat	−0.144 (−0.322, 0.035) 0.115	0.042 (−0.018, 0.102) 0.172	0.021 (−0.036, 0.078) 0.476
Fish	−0.210 (−0.449, 0.029) 0.086	−0.073 (−0.159, 0.013) 0.097	−0.091 (−0.172, −0.010) 0.028
Nuts	−0.292 (−0.464, −0.119) <0.001	−0.142 (−0.200, −0.084) <0.001	−0.159 (−0.213, −0.104) <0.001
Legumes	−0.144 (−0.365, 0.078) 0.204	−0.095 (−0.162, −0.028) 0.0058	−0.099 (−0.164, −0.035) 0.003
Whole grains	−0.029 (−0.209, 0.151) 0.753	−0.161 (−0.218, −0.104) <0.001	−0.148 (−0.202, −0.093) <0.001
Vegetables	−0.122 (−0.305, 0.062) 0.196	−0.162 (−0.224, −0.100) <0.001	−0.158 (−0.216, −0.099) <0.001
Fruit	−0.251 (−0.419, −0.082) 0.004	−0.128 (−0.185, −0.072) <0.001	−0.142 (−0.196, −0.089) <0.001
Model 2^b^			
Alcohol	−0.673 (−1.025, −0.321) <0.001	−0.156 (−0.265, −0.048) 0.005	−0.201 (−0.305, −0.097) <0.001
Monounsaturated to saturated fat ratio	−0.246 (−0.427, −0.065) 0.008	−0.158 (−0.219, −0.096) <0.001	−0.167 (−0.225, −0.108) <0.001
Red and processed meat	−0.184 (−0.367, −0.002) 0.049	−0.049 (−0.110, 0.013) 0.124	−0.064 (−0.123, −0.005) 0.033
Fish	−0.208 (−0.447, 0.031) 0.089	−0.070 (−0.156, 0.016) 0.111	−0.089 (−0.170, −0.008) 0.031
Nuts	−0.273 (−0.446, −0.099) 0.002	−0.182 (−0.241, −0.123) <0.001	−0.191 (−0.247, −0.135) <0.001
Legumes	−0.171 (−0.395, 0.052) 0.134	−0.091 (−0.158, −0.024) 0.008	−0.097 (−0.161, −0.032) 0.003
Whole grains	0.015 (−0.167, 0.196) 0.875	−0.172 (−0.230, −0.115) <0.001	−0.151 (−0.207, −0.096) <0.001
Vegetables	−0.127 (−0.310, 0.057) 0.177	−0.165 (−0.227, −0.103) <0.001	−0.159 (−0.217, −0.100) <0.001
Fruit	−0.238 (−0.411, −0.065) 0.007	−0.132 (−0.189, −0.075) <0.001	−0.145 (−0.200, −0.091) <0.001
Model 3^c^			
Alcohol	−0.491 (−0.847, −0.136) 0.007	−0.074 (−0.182, 0.035) 0.184	−0.114 (−0.217, −0.010) 0.032
Monounsaturated to saturated fat ratio	−0.116 (−0.294, 0.062) 0.200	−0.109 (−0.168, −0.050) <0.001	−0.106 (−0.162, −0.050) <0.001
Red and processed meat	−0.163 (−0.339, 0.013) 0.070	0.010 (−0.049, 0.069) 0.733	−0.013 (−0.069, 0.043) 0.650
Fish	−0.167 (−0.396, 0.062) 0.153	−0.030 (−0.112, 0.052) 0.473	−0.046 (−0.123, 0.031) 0.239
Nuts	−0.158 (−0.328, 0.011) 0.068	−0.065 (−0.123, −0.006) 0.030	−0.075 (−0.130, −0.020) 0.008
Legumes	−0.095 (−0.311, 0.121) 0.389	−0.015 (−0.080, 0.050) 0.647	−0.019 (−0.081, 0.043) 0.540
Whole grains	0.033 (−0.141, 0.208) 0.709	−0.091 (−0.146, −0.035) 0.001	−0.073 (−0.126, −0.020) 0.007
Vegetables	−0.053 (−0.237, 0.131) 0.571	−0.052 (−0.113, 0.009) 0.098	−0.049 (−0.107, 0.009) 0.099
Fruit	−0.060 (−0.234, 0.114) 0.498	−0.034 (−0.089, 0.021) 0.227	−0.040 (−0.093, 0.013) 0.135

CI, confidence interval; AMED, Adherence to Mediterranean Diet. ^a^Model 1 was non-adjusted model; ^b^Model 2 was adjusted for gender, race, age; ^c^Model 3 was adjusted for the following variables: gender, race, age, physical activity (MET), health insurance, sleep disorders, diabetes, education, hypertension, marital status, BMI, and alcohol use. *p* < 0.05 presents significant difference.

#### 3.3.2 Non-cancer group and total population

For the non-cancer group, nuts, whole grains, and the monounsaturated to saturated fat ratio consistently showed significant inverse associations with fatigue in all models (Table 3). At the same time, alcohol and fruit were only significantly correlated in Models 1 and 2. In Model 3, the monounsaturated to saturated fat ratio showed the strongest inverse association with a *β* of −0.109 (95% CI: −0.168, −0.050, *p* < 0.001), while alcohol was not significantly associated with fatigue (*p* = 0.184). Red and processed meat did not show a significant association in any model.

In the overall population, patterns similar to those observed in the cancer group were present (Table 3). Alcohol and nuts continued to show significant inverse associations with fatigue. In Model 3, alcohol (*β* = −0.114, 95% CI: −0.217, −0.010, *p* = 0.032) and the monounsaturated to saturated fat ratio (*β* = −0.106, 95% CI: −0.162, −0.050, *p* < 0.001) were significantly associated with lower fatigue. Whole grains were significantly correlated in all Models; fruit had significant associations in Models 1 and 2, while red and processed meat showed significance only in Model 2. Fish and legumes did not have significant effects in the fully adjusted model.

### 3.4 Subgroup analysis in the cancer group

In the fully adjusted model, diabetes, education level and type of cancer were significant factors moderating the relationship between AMED and fatigue (*p* < 0.05) (Table 4). The effect sizes for diabetics and non-diabetics were *β* = −0.196 and *β* = −0.012, respectively, with an interaction *p*-value of 0.010. Educational level also showed a significant interaction (*p* = 0.023), with effect sizes ranging from −0.063 for college graduates or higher to −0.204 for high school graduates or GED holders. In the cancer type subgroup, a significant inverse association was observed in breast cancer patients (*β* = −0.207, 95% CI: −0.361, −0.052, *p* = 0.009).

**Table 4:** Subgroup analysis of the relationship between AMED score and fatigue in the cancer group.

Stratified	*β* (95%CI)^a^	*p*-value	*p* for interaction
Gender			0.715
Male	−0.072 (−0.151, 0.007)	0.075	
Female	−0.053 (−0.123, 0.017)	0.140	
Age			0.182
<60	−0.117 (−0.216, −0.019)	0.020	
≥60	−0.039 (−0.103, 0.025)	0.230	
Race			0.509
Mexican American	−0.277 (−0.643, 0.088)	0.137	
Other Hispanic	0.076 (−0.587, 0.739)	0.821	
Non-Hispanic White	−0.051 (−0.111, 0.010)	0.103	
Non-Hispanic Black	−0.182 (−0.425, 0.061)	0.142	
Other Race – Including Multi-Racial	−0.132 (−0.450, 0.187)	0.418	
Education			0.023
Less than 9th grade	0.143 (−1.297, 1.582)	0.846	
9-11th grade (12th grade with no diploma)	−0.065 (−0.390, 0.261)	0.698	
High school graduate/GED or equivalent	−0.204 (−0.320, −0.089)	0.001	
Some college or AA degree	0.036 (−0.065, 0.138)	0.484	
College graduate or above	−0.063 (−0.148, 0.022)	0.145	
Marital status			0.925
Married/Living with partner	−0.059 (−0.123, 0.006)	0.074	
Widowed/Divorced/Separated	−0.072 (−0.172, 0.028)	0.161	
Never married	−0.018 (−0.297, 0.260)	0.898	
BMI			0.120
Underweight	0.563 (−0.686, 1.812)	0.378	
Normal weight	0.058 (−0.074, 0.190)	0.392	
Over weight	−0.070 (−0.161, 0.021)	0.133	
Obesity	−0.096 (−0.180, −0.011)	0.026	
Alcohol use			0.590
Yes	−0.065 (−0.141, 0.011)	0.093	
No	−0.037 (−0.115, 0.041)	0.354	
Hypertension			0.856
Yes	−0.072 (−0.145, 0.001)	0.053	
No	−0.063 (−0.140, 0.014)	0.110	
Diabetes			0.010
Yes	−0.196 (−0.332, −0.060)	0.005	
No	−0.010 (−0.070, 0.050)	0.743	
Health insurance			0.660
Yes	−0.081 (−0.135, −0.027)	0.003	
No	0.240 (−1.245, 1.726)	0.752	
Physical activity (MET, min/week)			0.637
Low	0.055 (−0.119, 0.228)	0.537	
Moderate	−0.040 (−0.170, 0.091)	0.554	
High	0.008 (−0.079, 0.095)	0.857	
Sleep disorder			0.659
Yes	−0.049 (−0.140, 0.042)	0.290	
No	−0.073 (−0.139, −0.008)	0.029	
Type of cancer^b^			0.049
Respiratory system	−0.758 (−2.656, 1.141)	0.435	
Head and neck	17.385 (−89.084, 123.854)	0.749	
Digestive System	0.171 (−0.173, 0.516)	0.330	
Breast cancer	−0.207 (−0.361, −0.052)	0.009	
reproductive system	0.005 (−0.110, 0.119)	0.936	
Urologic tumors	0.221 (−0.561, 1.002)	0.580	
Hematologic	−1.220 (−10.147, 7.708)	0.789	
Endocrine System	0.350 (−1.846, 2.545)	0.755	
Skin	−0.039 (−0.139, 0.061)	0.449	
Musculoskeletal system	−5.793 (−35.895, 24.308)	0.706	
Nervous system	28.836 (−126.461, 184.133)	0.716	
Other cancers	0.035 (−0.452, 0.521)	0.888	
Do not know	6.150 (−28.191, 40.490)	0.726	
Number of cancer type			0.852
1	−0.071 (−0.128, −0.014)	0.015	
2	−0.048 (−0.296, 0.200)	0.705	
3	0.261 (−1.908, 2.429)	0.814	
≥4	−0.405 (−1.108, 0.298)	0.260	

CI, confidence interval; AMED, Alternative Mediterranean Diet; MET, Metabolic Equivalent Task; BMI, Body Mass Index. *p* < 0.05 presents significant difference. ^a^Adjust for gender, race, age, physical activity (MET), health insurance, sleep disorders, diabetes, education, hypertension, marital status, BMI, and alcohol use. All the models are not adjusted for the variable itself in each stratification. ^b^Cancers are classified according to the anatomical system or site where they occur, into 13 categories.

## 4 Discussion

In this cross-sectional survey study with a nationally representative sample, a significant negative association was found between Mediterranean dietary adherence (AMED score) and fatigue in either the full or unadjusted model, particularly in the cancer population, suggesting that this dietary pattern may contribute to reducing cancer-related fatigue. Further analysis revealed that, the trend association between fatigue and AMED quartiles was also statistically significant, particularly within the cancer population. The results of the analyses of the Mediterranean diet components specifically showed a significant effect of alcohol intake on fatigue in the cancer population after adjusted for all the covariates in Table 1. Within cancer group, subgroup analyses and interaction results showed that diabetes, education level and type of cancer significantly modified the magnitude of the effect of AMED on fatigue.

In the present study, a significant negative correlation was identified between adherence to the Mediterranean diet and fatigue, both among cancer and non-cancer groups. This correlation was particularly pronounced among cancer participants. This finding aligns with previous research. For example, Brenton J. Baguley et al. conducted a study on Mediterranean diet (MED-diet) interventions in prostate cancer patients. Their 12-week MED-diet intervention significantly improved CRF, with mean changes (95% CI) of +4.8 (0.0, 9.8) at 8 weeks and + 7.2 (2.2, 12.0) at 12 weeks, respectively, compared to the conventional group (27). This benefit of the Mediterranean diet may be attributable to its anti-inflammatory and antioxidant properties. Existing studies suggest that inflammation (50) and oxidative stress (51) are key mechanisms underlying CRF. The Mediterranean diet is distinguished by a substantial consumption of plant-based foods, including fruits, vegetables, legumes, cereals, and nuts; olive oil as the primary source of fat (rich in monounsaturated fatty acids); moderate consumption of dairy products, fish, and poultry; minimal red meat intake; and moderate alcohol consumption. This dietary structure not only ensures a balanced nutritional intake required by cancer patients but is also abundant in antioxidants (e.g., polyphenols, vitamin E) and anti-inflammatory components (e.g., omega-3 fatty acids). In some interventional studies, the Mediterranean diet has also been observed to reduce inflammatory markers in cancer patients (52, 53). Additionally, a high intake of vegetables and fruits in the Mediterranean diet provides adequate amounts of carotenoids for cancer patients. A study by Amber S. Kleckner et al. utilizing NHANES population data, investigated the relationship between serum carotenoids and cancer-related fatigue. According to their findings, there was a 6.8–9.9% decrease in the probability of exhaustion for every standard deviation increase in blood carotenoid concentration, suggesting a potential correlation between higher serum carotenoid levels and lower fatigue (34). Nonetheless, given that these indicators were not directly measured in the present study, it is impossible to draw causal conclusions. Therefore, it is recommended that future studies incorporate relevant laboratory indicators to provide more conclusive evidence.

In the baseline analysis, although the fatigue scores within the two groups showed no statistically significant difference overall, further investigation revealed a statistically significant difference in the distribution of fatigue characteristics between the cancer and non-cancer participants. In particular, the cancer group experienced a more significant percentage of severe exhaustion (3 scores), whereas the general population experienced a higher percentage of minor fatigue (1 score). This aligns with the findings of some extant studies. Previous studies have shown that around 30 to 60% of cancer patients experience moderate to severe fatigue during treatment (8, 54, 55). However, the prevalence of moderate fatigue in the general adult population is about 14.6%, while the prevalence of severe fatigue is only 6.1% (56). In non-cancer populations, fatigue (e.g., physiologic fatigue) can be effectively alleviated by ensuring proper rest and replenishing energy stores (57). In contrast, CRF tends to be more severe and complicated to alleviate with rest (58).

Detailed analyses of the relationship between individual nutrients in the Mediterranean diet and CRF showed that moderate alcohol consumption was consistently and significantly associated with reduced fatigue in all models. Moderate alcohol consumption (especially red wine) is a part of the traditional Mediterranean diet and is considered beneficial. The mechanisms underlying the potential effects of small or moderate alcohol consumption on cancer-related fatigue may be attributed to the antioxidant and anti-inflammatory effects of alcohol. Some studies suggested antioxidant and anti-inflammatory activities among the bioactive phenolic compounds in red wine (59, 60). It is important to note that the present study did not measure inflammatory biomarkers. Consequently, the study was unable to assess these mechanisms directly. In this context, the role of alcohol in oncology patients remains complex. While moderate red wine consumption, as part of the Mediterranean diet, may exert potential benefits through antioxidant properties (61), it is critical to recognize that alcohol is also a well-established risk factor for cancer. Its carcinogenic effects have been linked to multiple mechanisms, including increased estrogen levels, disruption of folate metabolism, stimulation of cell proliferation, facilitation of carcinogen transport, inhibition of DNA methylation, and the metabolism of carcinogen precursors (62–64). Notably, continued alcohol consumption after a cancer diagnosis, particularly excessive consumption, has been associated with reduced quality of life in cancer survivors (65, 66), an increased risk of cancer recurrence, and decreased overall survival (67–69). These contradictory effects highlight the need for further research to clarify whether any potential benefits of moderate red wine consumption outweigh the established risks in oncology populations.

In our study, we analyzed in depth the relationship between adherence to the Mediterranean diet (AMED) and fatigue, finding that diabetes diagnosis, education level and type of cancer, significantly moderated this relationship. Specifically, diabetic patients benefited more from the Mediterranean diet than non-diabetic patients, showing a significant reduction in fatigue. Fatigue is a prevalent clinical symptom among individuals with diabetes (70), cancer patients with diabetes may experience compounded fatigue from both diabetes and cancer. A previous study that included 674 breast cancer patients also showed a significant increase in fatigue in diabetics compared to non-diabetics (71). The Mediterranean diet, abundant in fruits, vegetables, nuts, whole grains, and olive oil, promotes weight loss and improves insulin sensitivity (72), thereby contributing to more efficient use of insulin in people with diabetes, improved blood glucose regulation, and potential benefits in reducing fatigue caused by fluctuations in blood glucose (73).

Additionally, education level also significantly moderated the relationship between AMED and CRF. Among high school graduates or GED certificate holders, AMED was significantly and negatively associated with fatigue, indicating that patients at this education level experienced a greater reduction in fatigue. According to previous research, higher-educated individuals are more likely to comprehend and adhere to dietary recommendations, such as the Mediterranean diet, which may have more substantial health benefits (74, 75).

The results of the subgroup analyses further indicated that cancer type played a significant moderating role in the relationship between AMED scores and fatigue, with a significant negative correlation observed especially in breast cancer patients. It is suggested that the Mediterranean diet may have potential benefits in alleviating fatigue in breast cancer patients. This finding aligns with the results of previous studies. For example, Zick et al. (76) demonstrated that a 3-month dietary intervention in 30 breast cancer patients, which emphasized a high intake of natural foods (vegetables, fruits, fish, whole grains) and omega-3 fatty acids, reduced fatigue in breast cancer survivors. A study investigating fatigue during chemotherapy in cancer patients (91% of whom had breast cancer) also found that adherence to the Mediterranean diet reduced fatigue to mild and moderate levels at weeks 4 and 8 compared to usual care (28). These benefits may also be attributed to the anti-inflammatory and antioxidant properties of the Mediterranean diet. Olive oil in the Mediterranean diet is rich in polyphenolic compounds, which have been shown in previous studies to reduce oxidative stress in breast cancer cells, thereby may reduce fatigue levels (77).

Overall, these results highlight the need for personalized dietary intervention. Future studies should further explore the relevant biological mechanisms and validate the effect of dietary intervention in different patient groups through stratified intervention trials to improve the study’s generalizability and clinical application value.

## 5 Strengths and limitations

Studies have previously investigated the potential of the Mediterranean diet to mitigate fatigue in cancer patients. However, most of these studies were pilot studies with small sample sizes. They focused on specific cancer populations, such as breast cancer and prostate cancer, since they are the most common cancers in women and men worldwide. Moreover, CRF is particularly burdensome in these populations. These factors may limit statistical efficacy and the generalizability of findings. In contrast, our study utilizes a large, nationally representative sample to examine the relationship between adherence to the Mediterranean diet and fatigue, particularly in cancer populations. Our results suggest that the Mediterranean diet may offer a promising approach to mitigating fatigue, supporting the potential role of nutritional interventions in addressing fatigue in these populations.

Despite providing valuable insights into the association between the Mediterranean diet and fatigue, this study has several limitations that warrant consideration. (1) Cross-Sectional Design: The cross-sectional nature of the study precludes establishing a causal relationship between AMED score and CRF. This design captures associations at a single point in time, but does not allow for the determination of cause and effect. Longitudinal or interventional studies would be required to establish causality. (2) Assessment of Fatigue with PHQ-9: Although the PHQ-9 has been widely used to assess depressive symptoms, its single item measuring fatigue has not been specifically validated for cancer-related fatigue. This could limit the accuracy of capturing the full scope of fatigue in cancer patients, potentially overlooking some nuances. Future studies should aim to use more comprehensive, cancer-specific fatigue measurement tools to better assess this complex symptom. (3) Potential Selection Bias: A substantial number of participants were excluded due to missing data on fatigue, cancer diagnosis, or AMED scores, which may result in a sample that is not fully representative of the general population, thereby introducing selection bias. (4) Self-Reported Data: The dependence on self-reported nutritional intake and fatigue assessments may be influenced by memory bias and social desirability bias, thereby compromising data accuracy. (5) Residual Confounding: Although the analysis controlled for multiple covariates, there may still be unmeasured or inadequately controlled confounding factors that could influence the observed associations. Future research should aim to investigate additional moderators to enhance understanding of the diet-fatigue relationship. (6) Limitations of the database: The NHANES database does not collect crucial information about the clinical stage, treatment status, and presence of metastases in cancer patients. As a result, we could not conduct further subgroup analyses to understand how these factors might influence patient differences. Therefore, although the present study provides preliminary evidence supporting the potential benefits of the Mediterranean diet in alleviating fatigue, it is essential to interpret its results with caution due to the aforementioned limitations.

## 6 Conclusion

In conclusion, the results of this study suggest that AMED score was significantly negatively correlated with fatigue, more pronounced in cancer patients. In addition, within the cancer cohort, we found that individuals with diabetes, higher educational levels, and breast cancer may benefit more, suggesting that dietary interventions should consider individual differences and needs. Finally, due to the limitations of the cross-sectional design, the present study was unable to draw causal conclusions regarding the association between adherence to the Mediterranean diet and CRF, and further studies are needed to verify this in the future.

## Data Availability

The datasets presented in this study can be found in online repositories. The names of the repository/repositories and accession number(s) can be found at: https://www.cdc.gov/nchs/nhanes/.

## Footnotes

^1^ http://www.rproject.org

^2^ http://www.empowerstats.com
