# Supplementary material for: Adherence to the Mediterranean diet is associated with lower cancer-related fatigue: a cross-sectional analysis from NHANES 2017–2020
Source: Front Nutr. 2025 Mar 19;12:1506055. doi: 10.3389/fnut.2025.1506055 (PMC11961423; doi:10.3389/fnut.2025.1506055)
Supplement: Supplementary file 1 [file Table_1.docx]

Supplementary Material

# Table S1 Number of patients with specific diagnoses and all types of cancer

| **Diagnosis** | **First diagnosis of cancer (total N=707)**  **n** | **Second cancer diagnosis (total N=71)**  **n** | **Third cancer diagnosis (total N=14)**  **n** | **More than three cancer diagnosis (total N=3)**  **n** |
| --- | --- | --- | --- | --- |
| Bladder | 16 | 2 | None | None |
| Blood | 2 | 1 | None | None |
| Bone | 5 | 1 | None | None |
| Brain | 3 | 2 | None | None |
| Breast | 104 | 6 | None | None |
| Cervix (cervical) | 36 | 1 | None | None |
| Colon | 39 | 6 | None | None |
| Esophagus (esophageal) | 4 | 1 | None | None |
| Kidney | 20 | 2 | None | None |
| Larynx/ windpipe | 1 | None | None | None |
| Leukemia | 5 | None | 1 | None |
| Liver | 2 | 1 | None | None |
| Lung | 18 | None | 3 | None |
| Lymphoma/ Hodgkins | 18 | 3 | None | None |
| Melanoma | 55 | 4 | None | None |
| Mouth/tongue/lip | 3 | None | None | None |
| Ovary (ovarian) | 13 | 2 | 1 | None |
| Pancreas (pancreatic) | 1 | None | None | None |
| Prostate | 100 | 9 | 1 | None |
| Rectum (rectal) | 4 | 2 | None | None |
| Skin (non-melanoma) | 87 | 7 | 2 | None |
| Skin (don't know what kind) | 45 | 9 | 2 | None |
| Stomach | 4 | None | None | None |
| Testis (testicular) | 3 | None | None | None |
| Thyroid | 25 | 3 | None | None |
| Uterus (uterine) | 31 | 4 | 1 | None |
| Other | 45 | 5 | 3 | None |
| Don't know | 18 | None | None | 3 |

# Table S2 Comparison of Fatigue Scores Between Cancer and Non-Cancer Groups: Stratified Z-Test Results

| **Fatigue score** | **Cancer (n=707)** | **Non-Cancer (n=5706)** | **z-value** | **p-value** |
| --- | --- | --- | --- | --- |
| 0 | 351 (49.65%) | 2824 (49.47%) | 0.078 | 0.938 |
| 1 | 209 (29.56%) | 1908 (33.42%) | -2.068 | 0.039 |
| 2 | 62 (8.77%) | 512 (8.97%) | -0.179 | 0.858 |
| 3 | 85 (12.02%) | 462 (8.13%) | 3.525 | <0.001 |

p< 0.05 presents significant difference.
